# Correction: The diagnostic performance of combined conventional cytology with smears and cell block preparation obtained from endoscopic ultrasound-guided fine needle aspiration for intra-abdominal mass lesions

**DOI:** 10.1371/journal.pone.0271369

**Published:** 2022-07-08

**Authors:** Nonthalee Pausawasdi, Penprapai Hongsrisuwan, Wipapat Vicki Chalermwai, Amna Subhan Butt, Kotchakon Maipang, Phunchai Charatcharoenwitthaya

The sixth author’s name is spelled incorrectly. The correct name is: Phunchai Charatcharoenwitthaya. The correct citation is: Pausawasdi N, Hongsrisuwan P, Chalermwai WV, Butt AS, Maipang K, Charatcharoenwitthaya P (2022) The diagnostic performance of combined conventional cytology with smears and cell block preparation obtained from endoscopic ultrasound-guided fine needle aspiration for intra-abdominal mass lesions. PLoS ONE 17(3): e0263982. https://doi.org/10.1371/journal.pone.0263982
