# Supplementary material for: The diagnostic performance of combined conventional cytology with smears and cell block preparation obtained from endoscopic ultrasound-guided fine needle aspiration for intra-abdominal mass lesions
Source: PLoS One. 2022 Mar 23;17(3):e0263982. doi: 10.1371/journal.pone.0263982 (PMC8942242; doi:10.1371/journal.pone.0263982)
Supplement: S1 Table — (DOCX) [file pone.0263982.s001.docx]

**S1 Table.** The **a**ntibody clones for immunohistochemistry staining

| **Antibody** | **Clone** | **Source** |
| --- | --- | --- |
| AE1/AE3 | AE1/AE3 | DAKO, CA, USA |
| CAM5.2 | B22.1&B23.1 | Thermo Fisher Scientific, MA, USA |
| CD45 | 2B11+PD7/26 | DAKO, CA, USA |
| CK7 | OV-TL12/30 | Cell Marque, CA, USA |
| CK20 | KS20.8 | Cell Marque, CA, USA |
| S-100 | polyclonal | DAKO, CA, USA |
| Vimentin | SP20 | Cell Marque, CA, USA |
| Desmin | D33 | DAKO, CA, USA |
| CDX-2 | ERP2764Y | Cell Marque, CA, USA |
| TTF-1 | 8G7G3/1 | Cell Marque, CA, USA |
| chromogranin A | LK2H10 | Thermo Fisher Scientific, MA, USA |
| synaptophysin | polyclonal | Cell Marque, CA, USA |
| HepPar1 | OCH1E5 | Cell Marque, CA, USA |
| AFP | polyclonal | Cell Marque, CA, USA |
| PAX8 | polyclonal | Cell Marque, CA, USA |
| RCC | PN-15 | Cell Marque, CA, USA |
| CD10 | 56C6 | Novoastra, Leica Biosystems, IL, USA |
| CD34 | QBEnd/10 | Cell Marque, CA, USA |
