# Supplementary material for: The diagnostic performance of combined conventional cytology with smears and cell block preparation obtained from endoscopic ultrasound-guided fine needle aspiration for intra-abdominal mass lesions
Source: PLoS One. 2022 Mar 23;17(3):e0263982. doi: 10.1371/journal.pone.0263982 (PMC8942242; doi:10.1371/journal.pone.0263982)
Supplement: S1 Data — (PDF) [file pone.0263982.s002.pdf]

NO.Total	Dermographic data				FNA data			Final diagnosis	
	Sex	Age	GI Problem	Indication	Needle No.	No. of pass	Suction	Final diagnosis	Dx based on
	0 male		0 Abd pain	0 Pan mass	0=19G	0= 1	0= yes	0= CA pan.	0= cyto. Result
	1 female		1 Jaundice	1 intraabd. LN	1=22G	1= 2	1= No	1=inflam/ reactive	1= cell block result
			2 Abd mass	2 Liver mass	2= 25G	2= 3		2=NET	2= Surgical specimen
			3 Wt. loss	3 CBD/Bile duct mass	3= other	3= 4		3= Metas	3= Clinical presentation
			4 Abn. LFT	4 GB mass		4= >=5		4= Lymphoma	
			5 Abn imaging	5 Bowel wall mass				5= other	
			6 other	6 Adrenal mass					
				7 unknown origin mass					
				8 other					
1	1	37	0,3,5	1	1	1	0	1	3
2	0	36	6 : anemia	5	1	3	0	4	0,1,3
3	1	49	0,5	1	1	3	0	3	0,3
4	1	60	0,5	: Retropentoneal ma	1	1	0	5	1,3
5	1	1	1,3,5	0	1	1	0	0	0,1,3
6	1	65	0,5	0	1	3	0	0	0, 3
7	0	62	5	1	1	3	0	1	0, 1, 3
8	1	49	3,5	5	1	3	0	5	0, 1, 2, 3
9	0	51	5	0	1	1	0	1	3
10	0	73	0	1	1	3	0	1	0, 1, 3
11	0	36	0,5	1	1	1	0	3	0, 1, 3
12	0	50	0,5	5	1	3	0	1	0, 1, 3
13	1	66	2,5	0	0	3	0	0	0, 1, 3
14	1	74	3, 5	0	1	2	0	0	0, 1, 3
15	1	76	3, 4	0	1	4	0	0	0, 1, 3
16	0	84	5	1	1	4	0	4	0, 1, 3
17	0	65	3, 5	1	1	1	0	1	3
18	0	48	3, 5	0	1	3	0	0	1, 3

19	0	53	0, 5	1	1	3	0	3	0, 1, 3
20	0	87	5	5	1	1	0	5	0, 1, 3
21	0	50	1	1	1	3	0	1	3
22	0	56	5	0	1	4	0	3	0, 1, 3
23	0	68	5	0	2	3	0	0	0, 3
24	0	64	5	0	1	1	0	1	0, 1, 3
25	1	71	2, 5	0	1	3	0	3	0, 1, 3
26	0	66	0	1, 2	1	3	0	3	0, 1, 3
27	0	63	5	0, 1	1	1	0	3	3
28	1	44	1	1	1	1	0	1	3
29	0	58	0,5	0	1	3	0	1	0, 1, 3
30	0	28	3	1	1	4	0	1	3
31	0	56	5	1	1	2	1	3	0, 1, 3
32	1	62	2	5	1	3	0	5	0, 1, 3
33	0	77	0	1	1	3	0	3	0, 1, 3
34	1	66	5	1, 4	1	3	0	3	0, 3
35	0	77	0, 5	5	1	3	0	1	3
36	1	65	5	0, 2	1	0	0	0	1, 3
37	1	77	5	0, 1	1	3	0	0	3
38	0	37	5	0	1	4	0	1	3
39	0	63	5	1, 5	1	1	0	3	3
40	1	60	0, 5	0	1	1	0	0	3
41	0	79	0,5	0	1	3	0	0	0, 1, 3
42	0	28	3, 5	1	1	3	0	1	3
43	0	64	0, 5	0, 2	1	1	0	0	0, 1, 3
44	1	68	0	1	1	3	0	3	0, 1, 3
45	0	28	5	1	1	3	0	1	0, 1, 3
46	0	51	1, 3, 4, 5	0	1	1	0	0	2, 3
47	0	52	0, 5	0	1	3	0	1	0, 1, 3
48	1	63	2, 5	0, 1	1	3	0	3	0, 1, 3
49	0	26	0, 5	1	1	1	0	1	0, 1, 3
50	0	60	5	0	1	3	0	3	0, 1, 3
51	0	57	0, 5	0	1	4	0	0	0, 3
52	0	25	5	0, 1	1	3	0	1	3
53	1	62	5	0	1	3	0	0	1, 3
54	0	63	0, 4, 5	0	1	0	0	3	3
55	1	64	1, 5	0	1	1	0	0	3

56	1	48	5	5	1	1	0	5	2, 3
57	0	74	5	0	1	1	0	1	3
58	1	74	0, 5	0	1	1	0	5	2, 3
59	1	52	0, 5	5	1	3	0	3	2, 3
60	1	62	1, 5	0	1	0	0	5	3
61	0	58	0, 5	1	1	1	0	1	1, 3
62	0	59	1, 3, 5	0	1	1	0	0	0, 3
63	1	47	5	5	1	1	0	5	2, 3
64	1	62	1, 5	1	1	0	0	5	2, 3
65	0	1	0	1	1	0	0	0	0, 1, 3
66	1	56	0, 5	0, 2	1	1	0	0	0, 1, 3
67	1	60	1, 5	0	1	1	0	0	0, 1, 3
68	0	75	1, 4, 5	1	1	0	0	0	2, 3
69	1	58	0, 5	0, 1	1	3	0	0	0, 3
70	1	73	0, 5	0	1	0	0	0	2, 3
71	0	37	0	0	1	3	0	0	2, 3
72	0	85	3, 5	0	1	4	0	0	0, 1, 3
73	0	53	0, 3, 5	0	1	3	0	0	0, 1, 3
74	1	32	0, 5	1	1	1	0	1	3
75	0	61	5	1	1	3	0	3	0, 1, 3
76	0	42	5	1	1	3	0	1	3
77	1	73	0, 3, 5	0	1	1	0	5	0, 1, 3
78	1	51	0, 3, 5	0	1	1	0	0	0, 1, 3
79	0	42	0, 3, 5	0	1	1	0	0	0, 1, 3
80	0	75	0, 3, 5	1	1	1	0	3	0, 1, 3
81	1	75	5	1	1	1	0	3	0, 1, 3
82	1	56	0, 5	0, 2	1	1	0	0	2, 3
83	1	73	1, 5	0	1	0	0	0	2, 3
84	0	37	5	0	1	3	0	0	0, 3
85	0	31	0, 3	0	1	1	0	1	0, 1, 3
86	1	68	0, 5	0	1	3	0	0	0, 1, 3
87	1	59	1, 4, 5	0	1	0	0	0	3
88	0	53	4, 5	0	1	1	0	1	0, 1, 3
89	1	61	0, 3	0	1	3	0	0	0, 3
90	1	59	1, 3, 5	0, 2	1	4	1	0	0,1 , 3
91	1	19	0, 5	0	1	0	0	3	3
92	0	74	1, 5	0, 1	1	0	0	3	3

93	0	45	5	1	1	0	0	1	3
94	1	50	5	5	1	1	0	3	0,1 ,3
95	1	63	3, 5	1	1	3	0	5	0,1 ,3
96	0	66	0, 5	0	1	1	0	0	0,1 ,3
97	1	77	5	0	1	1	0	1	3
98	0	61	1, 5	1	1	1	0	3	0, 3
99	0	58	5	0	1	3	0	0	0, 3
100	0	74	1, 5	0, 1	1	1	0	3	3
101	1	56	0, 5	0	1	1	0	0	0,1 ,3
102	1	61	5	0	1	4	0	3	3
103	0	33	1, 3, 5	0	1	1	0	0	0,1 ,3
104	1	77	0	0	1	1	0	1	0, 3
105	1	33	5	1	1	1	0	1	3
106	1	79	0, 1, 5	1	1	1	0	3	0,1 ,3
107	1	56	5	0	1	1	0	0	2, 3
108	1	64	0, 3, 5	0	2	1	0	0	0,1 ,3
109	1	80	5	5	1	1	0	3	3
110	0	52	0, 5	0	1	1	0	1	1, 3
111	1	42	5	2	1	0	0	5	3
112	0	61	5	1	1	0	0	3	0,1 ,3
113	1	53	5	1	1	3	0	3	0,1 ,3
114	0	58	3, 5	0	1	3	0	0	0,1 ,3
115	1	61	0	1	1	0	0	1	3
116	0	57	5	1	1	3	0	3	1, 3
117	1	68	6	7	1	3	0	3	0,1 ,3
118	0	46	3, 5	0, 2	1	1	0	0	1, 3
119	1	75	1, 3, 5	0	1	1	0	0	1, 3
120	0	55	0	0	2	3	0	1	1, 3
121	1	60	0	0	1	1	0	0	3
122	0	54	5	1	1	1	0	1	0,1 ,3
123	0	60	0, 5	0	1	1	0	0	0,1 ,3
124	1	68	5	1	1	1	0	3	3
125	1	60	0, 5	0	1	3	0	0	3
126	0	56	0, 5	1	1	3	0	3	1, 3
127	0	58	0, 5	0	1	3	0	1	3
128	0	54	1, 5	1	1	1	0	3	0,1 ,3
129	0	56	3, 5	1	1	1	0	3	3

130	1	66	5	0	1	0	0	3	1, 3
131	1	56	5	1	1	1	0	3	0,1 ,3
132	1	72	0, 5	0	1	1	0	0	3
133	0	55	3, 5	0	1	3	0	0	0,1 ,3
134	0	53	5	5	1	1	0	5	3
135	0	51	0, 5	0, 2	1	0	1	0	0,1 ,3
136	0	77	4, 5	0	1	3	0	3	0,1 ,3
137	1	56	0, 3, 5	1	1	0	0	3	0,1 ,3
138	0	77	0, 3, 5	0	1	1	0	3	0,1 ,3
139	0	49	0, 3, 5	0	1	1	0	1	3
140	1	62	5	1	1	1	0	3	0,1 ,3
141	0	54	3, 5	1	1	1	0	1	0, 1, 3
142	1	62	5	1	1	2	0	3	0
143	1	50	5	1	1	2	0	3	0, 1, 3
144	0	66	0, 5	1	1	2	0	3	0, 1, 3
145	0	42	5	1	1	2	0	1	3
146	0	44	5	0	1	0	0	1	3
147	0	44	5	0	1	0	0	1	3
148	0	46	5	1	1	1	0	3	0, 1, 3
149	1	66	0, 3, 5	0	1	1	0	0	0, 1, 3
150	1	32	0, 5	1	1	1	0	1	0, 1, 3
151	0	23	0, 5	0, 1	1	2	0	0	2, 3
152	1	71	5	0	1	1	0	0	0, 1, 3
153	0	57	0, 5	0	1	0,2	0	1	0, 1, 3
154	1	46	0, 5	1	1	3	0	1	0, 1, 3
155	1	64	5	0	1	1	0	0	0, 1, 3
156	1	86	5	1	1	1	0	4	2, 3
157	1	74	1, 5	0	1	1	0	0	0, 1, 3
158	0	72	0, 5	0	1	2	0	0	3
159	0	31	0, 5	0	1	0	0	2	0, 1
160	0	32	0, 1, 3, 5	1	1	1	0	0	0, 1, 3
161	1	59	0, 3, 5	1	0	4	0	4	1, 3
162	0	65	5	1	1	0	0	3	1, 3
163	0	53	3, 5	0	1	0	0	0	3
164	0	55	5	7	1	1	0	4	0,1 ,3
165	0	55	5	7	1	0	0	4	0,1 ,3
166	1	55	5	1	1	0	0	0	2, 3
