# Supplementary material for: The diagnostic performance of combined conventional cytology with smears and cell block preparation obtained from endoscopic ultrasound-guided fine needle aspiration for intra-abdominal mass lesions
Source: PLoS One. 2022 Mar 23;17(3):e0263982. doi: 10.1371/journal.pone.0263982 (PMC8942242; doi:10.1371/journal.pone.0263982)
Supplement: Attachment — Submitted filename: Response to reviewers.RV2.docx [file pone.0263982.s004.docx]

Dear Editor,

We would like to thank the editor and both reviewers for reviewing our manuscript and providing constructive criticisms. As suggested, we have revised the manuscript point-by-point in response to the reviewers’ questions.

**Reviewers' comments:**

**Reviewer #1:** The authors have assessed the issues clarifying doubts and appropriately modifying the paper, therefore in my opinion the article can be submitted for publication.

***Response:*** We are grateful for the reviewer’s input which helps improve the quality of our manuscript. Your time is greatly appreciated.

**Reviewer #2:** I would like to thank the authors for all the answers to my comments and the revision of the manuscript. In my opinion there are still some issues that need to be addressed before publication:

***Response:*** We thank the reviewer for taking the time to review our manuscript and providing helpful comments. We have revised our manuscript to address the reviewer’s comments, and a point-by-point response to the specific questions is provided.

-The word "histology" is still often used directly in place of “cell block preparation”. In my opinion, the terminology must be checked through all the text (considering also the abstract and the tables).

***Response:*** Thank you for pointing this out. To make the terminology consistent throughout the article, we have changed the terminology regarding the tissue processing method (cytology, cytological evaluation, histology, cell block preparation) throughout the manuscript. Changes have been made to all sections, including the manuscript title, abstract, introduction, materials and methods, results, discussion, conclusion, tables, and the figure legend.

The manuscript title has been changed to ***“The diagnostic performance of combined conventional cytology with smears and cell block preparation obtained from endoscopic ultrasound-guided fine needle aspiration for intra-abdominal mass lesions”****.*

The word “cytological analysis” was replaced by “cytology” or “conventional cytology” or “cytologic smears” when appropriate.

The word “histology” was replaced by “cell block preparation” or “cell block preparation for histological assessment” when appropriate.

-Line 31 and others: ”combined cytological”: you should change it to “cytology” or “conventional cytology” through all the text.

***Response:*** Changes have been made as suggested. “combined cytological….” was changed to “combined conventional cytology and cell block preparation…..”

-Lines 127-130: “Three hundred and thirty-five patients underwent EUS-FNA for intra-abdominal mass lesions during the study period. One hundred and forty-five patients were excluded due to cystic lesions. Specimens were not available for review in 24 cases; thus, 166 patients were included in the study.” : you should report this part in the result section.

***Response:*** Changes have been made as suggested. Lines 127-130 were moved to the result section.

-Line 150: “ROSE is not available”: “ROSE was not available”.

***Response:*** Change has been made as suggested.

-Lines 159-166: “The immunohistochemical stains…”: you should mention the clones you used.

***Response:*** A summary of antibodies and clones used in this study has been put together in Supplementary table 1 (see below). The following sentence, “The information about antibody clones are provided in Supplementary Table 1” was added to the text under the Materials & Methods section, subheading “Cytological smears and cell block preparation”.

**Supplementary Table 1.** The antibody clones for immunohistochemistry staining

-Line 178 “(Pap)”: in my opinion you should use either the entire word or the abbreviation.

***Response:*** Pap was changed to Papanicolaou.

-Table 2, “Surgical pathology” column: you should check the concordance with the data reported in the text.

***Response:*** The concordance between the data in table 2 and the text was reviewed. Table 2 has been revised as suggested.

-Table 3: you should delete the “%” signs.

***Response:*** “%” signs were removed.

-Table 4: you should delete the word “abbreviation”.

***Response:*** The word “abbreviation” below Table 4 was removed.

-Line 290: where does the data “13 patients” come from?

***Response:*** We intended to state that in cases of surgical pathology proven malignancy, the diagnostic accuracy of EUS-FNA was 100%. The number of patients with surgical pathology was 16, not 13. “13” was a typo. However, we elected to remove this sentence as the number of patients with surgical pathology proven malignancy was quite limited, as mentioned in the study limitations, and added limited value.

-Lines 292-293, “Of 86 patients without the diagnosis of malignancy based on cytological evaluation (inadequate = 28, negative for malignancy = 58)”: you should report in the text the results of the FNAB of all the patients in this study.

***Response:*** Of 86 patients who had either inadequate cytology or cytological diagnosis of negative for malignancy, 58 patients had adequate specimens for cell block preparation. Of these, 8 patients (9.3%) were found to have malignancy, including pancreatic and metastatic cancer. Other diagnoses included tuberculous lymphadenitis (n = 10), reactive lymph nodes (n = 9), abscess (n = 3), GIST (n = 1), benign pancreatic tissue (n = 1). The diagnoses could not be made by cell block evaluation in 26 patients. Among these, malignancy (n = 16), tuberculosis (n = 6), and other benign conditions (n = 4) were discovered during clinical and imaging follow-up. These results have been added as suggested.
